# To Remove Nitrate of Silver Stains from the Fingers

**Published:** 1893-02

**Authors:** 


TO REMOVE NITRATE OF SILVER STAINS FROM THE FINGERS.—A correspondent of the *Scientific American* gives the following harmless process: First paint the blackened parts with tincture of iodine, let remain until the black becomes white. The skin will then be red, but by applying ammonia the iodine will be bleached, leaving white instead of black stains of nitrate of silver.

